# Mode equivalence and acceptability of tablet computer-, interactive voice response system-, and paper-based administration of the U.S. National Cancer Institute’s Patient-Reported Outcomes version of the Common Terminology Criteria for Adverse Events (PRO-CTCAE)

**DOI:** 10.1186/s12955-016-0426-6

**Published:** 2016-02-19

**Authors:** Antonia V. Bennett, Amylou C. Dueck, Sandra A. Mitchell, Tito R. Mendoza, Bryce B. Reeve, Thomas M. Atkinson, Kathleen M. Castro, Andrea Denicoff, Lauren J. Rogak, Jay K. Harness, James D. Bearden, Donna Bryant, Robert D. Siegel, Deborah Schrag, Ethan Basch, on behalf of the National Cancer Institute PRO-CTCAE Study Group

**Affiliations:** Department of Health Policy and Management, University of North Carolina at Chapel Hill, Campus Box 7411, Chapel Hill, NC 27599 USA; Division of Health Sciences Research, College of Medicine, Mayo Clinic, 13400 E. Shea Blvd, Scottsdale, AZ 85259 USA; Division of Cancer Control and Population Sciences, Outcomes Research Branch, National Cancer Institute, 9609 Medical Center Drive, East Tower, Suite 3-448, Rockville, MD 20850 USA; Department of Symptom Research, The University of Texas M. D. Anderson Cancer Center, 1400 Pressler Street, Unit 1450, Houston, TX 77030 USA; Department of Psychiatry and Behavioral Sciences, Memorial Sloan Kettering Cancer Center, 641 Lexington Avenue, 7th Floor, New York, NY 10022 USA; Cancer Therapy Evaluation Program, National Cancer Institute, 9609 Medical Center Drive, MSC 9737, Bethesda, MD 20892 USA; Department of Epidemiology and Biostatistics, Memorial Sloan Kettering Cancer Center, 485 Lexington Avenue, 2nd Floor, New York, NY 10017 USA; The Center for Cancer Prevention and Treatment, St. Joseph Hospital of Orange, 1100 West Stewart Drive, Orange, CA 92868 USA; Gibbs Cancer Center and Research Institute, Spartanburg Regional Healthcare System, 101 East Wood Street, Spartanburg, SC 29303 USA; The Cancer Program of Our Lady of the Lake and Mary Bird Perkins, 1950 Essen Lane, Baton Rouge, LA 70809 USA; Hartford Hospital-Helen and Harry Gray Cancer Center, 85 Retreat Avenue, Hartford, CT 06106 USA; Dana Farber Cancer Institute, 450 Brookline Ave, D-1008, Boston, MA 02215 USA; Department of Medicine, University of North Carolina at Chapel Hill, Campus Box 7305, Chapel Hill, NC 27599 USA

**Keywords:** PRO-CTCAE, Patient-Reported Outcomes, Symptoms, Adverse Events, Mode of Administration, Interactive Voice Response System

## Abstract

**Background:**

PRO-CTCAE is a library of items that measure cancer treatment-related symptomatic adverse events (NCI Contracts: HHSN261201000043C and HHSN 261201000063C). The objective of this study is to examine the equivalence and acceptability of the three data collection modes (Web-enabled touchscreen tablet computer, Interactive voice response system [IVRS], and paper) available within the US National Cancer Institute (NCI) Patient-Reported Outcomes version of the Common Terminology Criteria for Adverse Events (PRO-CTCAE) measurement system.

**Methods:**

Participants (*n* = 112; median age 56.5; 24 % high school or less) receiving treatment for cancer at seven US sites completed 28 PRO-CTCAE items (scoring range 0–4) by three modes (order randomized) at a single study visit. Subjects completed one page (approx. 15 items) of the EORTC QLQ-C30 between each mode as a distractor. Item scores by mode were compared using intraclass correlation coefficients (ICC); differences in scores within the 3-mode crossover design were evaluated with mixed-effects models. Difficulties with each mode experienced by participants were also assessed.

**Results:**

103 (92 %) completed questionnaires by all three modes. The median ICC comparing tablet vs IVRS was 0.78 (range 0.55–0.90); tablet vs paper: 0.81 (0.62–0.96); IVRS vs paper: 0.78 (0.60–0.91); 89 % of ICCs were ≥0.70. Item-level mean differences by mode were small (medians [ranges] for tablet vs. IVRS = −0.04 [−0.16–0.22]; tablet vs paper = −0.02 [−0.11–0.14]; IVRS vs paper = 0.02 [−0.07–0.19]), and 57/81 (70 %) items had bootstrapped 95 % CI around the effect sizes within +/−0.20. The median time to complete the questionnaire by tablet was 3.4 min; IVRS: 5.8; paper: 4.0. The proportion of participants by mode who reported “no problems” responding to the questionnaire was 86 % tablet, 72 % IVRS, and 98 % paper.

**Conclusions:**

Mode equivalence of items was moderate to high, and comparable to test-retest reliability (median ICC = 0.80). Each mode was acceptable to a majority of respondents. Although the study was powered to detect moderate or larger discrepancies between modes, the observed ICCs and very small mean differences between modes provide evidence to support study designs that are responsive to patient or investigator preference for mode of administration, and justify comparison of results and pooled analyses across studies that employ different PRO-CTCAE modes of administration.

**Trial registration:**

NCT Clinicaltrials.gov identifier: NCT02158637

## Background

The US National Cancer Institute (NCI) initiated development of a patient-reported outcome (PRO) measurement system for quantifying symptomatic adverse events in cancer clinical trials [1]. This system is intended to complement the existing long-standing approach to capturing investigator-reported adverse events using the Common Terminology Criteria for Adverse Events (CTCAE). Although the CTCAE provides a standard method for clinician grading of adverse effects, additional evaluation from the patient perspective is warranted since approximately 10 % of the adverse effects listed in the CTCAE are subjective symptoms that can be best evaluated by gathering information directly from patients. A recent systematic review confirms that clinicians often underestimate the incidence, severity and distress of the symptoms experienced by cancer patients [2]. In response to these challenges, the NCI has recently developed and validated the Patient-Reported Outcomes version of the Common Terminology Criteria for Adverse Events (PRO-CTCAE) [1, 3]. The PRO-CTCAE measurement system is comprised of a library of questions evaluating the various attributes (e.g., the presence, frequency, severity, and interference with usual activities) of 78 symptoms drawn from the CTCAE [3]. The PRO-CTCAE item library includes items that capture the full range of symptomatic treatment effects that may be experienced across a variety of disease sites and cancer treatment modalities. For more information about PRO-CTCAE and for permission to use, visit http://healthcaredelivery.cancer.gov/pro-ctcae/.

A survey of oncology clinical trialists and NCI representatives identified that an essential feature of the PRO-CTCAE measurement system is the capacity to administer items to patients via a variety of modes including tablet/personal computer, Interactive Voice Response System (IVRS; i.e., automated telephone questionnaire), and paper [4]. In response, a PRO-CTCAE software system was developed to allow assessment via these three modes in order to enhance its use across a variety of study contexts and populations, including individuals with limited literacy, limited access to the internet or telephone, or sensory impairments. However, to have confidence in the validity of the data collected using these different modes and to permit pooled analyses when different modes are used within and between studies, evidence is needed that individuals will provide the same responses to a PRO-CTCAE item regardless of which mode of administration is used.

A substantial number of studies of other PRO measures have evaluated the equivalence of paper vs. screen-based (tablet, laptop/desktop computer, or small handheld device) administration, across many domains and populations, and meta-analysis of these studies confirms high levels of reliability when paper-based and screen-based administration is compared [5, 6]. However fewer studies have evaluated the equivalence of visual formats (e.g., paper and screen) and aural formats such as IVRS [6–13]. The adaptations made to PRO measures to migrate from a visual format to IVRS are classified as a moderate level of modification by research guidelines [14], and thus formal quantitative evaluation of mode equivalence and an assessment of user satisfaction and usability testing are recommended. Further, in order to allow for multiple modes within a single study or to conduct pooled analyses of studies using different modes, evidence to support equivalence across modes of administration is crucial [15]. Usability testing of the Web-enabled touchscreen tablet computer and IVRS modes of administration of the PRO-CTCAE system were conducted as part of a larger study of patient and clinician usability [16]. The purpose of this study was to examine the between-mode equivalence and the relative acceptability of the three available modes of PRO-CTCAE administration in a diverse sample of patients undergoing cancer treatment. This study was conducted as a nested study within a large validation study of the English language version of PRO-CTCAE (clinicaltrials.gov identifier NCT02158637) [3].

## Methods

### Setting and sample

Adult patients with a solid tumor or hematologic malignancy, initiating or currently receiving chemotherapy, radiation therapy, or both, at one of three U.S.-based cancer centers and four community oncology practices in the U.S. NCI Community Cancer Centers Program (NCCCP) were eligible to participate in this study. The seven sites were: Dana-Farber Cancer Institute, Boston, MA; Memorial Sloan Kettering Cancer Center, New York, NY; University of Texas M. D. Anderson Cancer Center, Houston, TX; Hartford Hospital - Helen and Harry Gray Cancer Center, Hartford, CT; Our Lady of the Lake and Mary Bird Perkins Cancer Center, Baton Rouge, LA; Gibbs Cancer Center, Spartanburg, SC; and St. Joseph Hospital of Orange, Orange, CA. Potential subjects were approached in clinical waiting areas and invited to participate in this study. All participants could read and comprehend English and were without clinically significant cognitive impairment based on site investigator judgment.

Enrollment was limited at the academic institutions to specific tumor sites including breast, head, neck, or esophageal cancer; metastatic prostate, bladder, lung, or colorectal cancer; lymphoma or myeloma; at community oncology practices; enrollment was open to all tumor sites. Study sites were selected to achieve sampling diversity with respect to educational attainment, as well as geographic, racial/ethnic, and socio-economic factors.

### Ethics, consent, and permissions

Institutional review board approval was obtained at all sites and at the NCI, and all participants completed written informed consent.

### PRO-CTCAE Item Library

The PRO-CTCAE item library is composed of 124 self-report items reflecting 78 symptomatic adverse events, with each adverse event assessed relative to one or more attributes, including: presence/absence (P), frequency (F), severity (S), and/or interference (I) with usual or daily activities. The PRO-CTCAE item library includes items that capture the full range of symptomatic treatment effects that may be experienced across the full range of cancer treatment modalities [1, 3]. We examined the mode equivalence of 28 items measuring 14 symptomatic adverse events, specifically: anxiety [F,S, I], sad or unhappy feelings [F,S,I], constipation [S], diarrhea [F], anorexia [S,I], nausea [F,S], vomiting [F,S], mouth or throat sores [S,I], shortness of breath [S,I], numbness/tingling in hands and feet [S,I], pain [F,S,I], rash [P], fatigue [S,I], and insomnia [S,I]. These items were chosen for this study, based on the high prevalence of these symptoms in persons undergoing cancer treatment, including investigational treatment [17, 18]. Items measuring frequency, severity, and interference with daily activities used a 0–4 rating scale (i.e., *frequency*: (0) never, (1) rarely, (2) occasionally, (3) frequently, (4) almost constantly*; severity*: (0) none, (1) mild, (2) moderate, (3) severe, (4) very severe; and *interference with daily activities*: (0) not at all, (1) a little bit, (2) somewhat, (3) quite a bit, (4) very much). The response options for *presence/absence* were (0) no or (1) yes. The standard recall period for all PRO-CTCAE items is the past 7 days.

### Study Design

Participants completed the PRO-CTCAE questionnaire in clinic by each of the three modes (Web-enabled touchscreen tablet computer, IVRS, and paper) in a single study visit lasting approximately 45–60 min. The order in which each mode was to be completed was determined by randomized crossover design, in which participants were assigned in equal numbers to one of six possible orders for completing the questionnaire in each of the three modes, so that order effects could be identified and controlled in the analysis. The PRO-CTCAE screen-based and IVRS questionnaires employ conditional branching. For example, if a patient responds “Never” (0) to the frequency item, the subsequent items for that symptom assessing severity or interference with daily activities are not asked, and in the analysis it is assumed the response to these items is “None” (0) or “Not at all” (0). The paper version of PRO-CTCAE presents all the items for each symptomatic AE and does not include a skip pattern. Therefore, in this study a respondent could be asked to complete as many as 28 items, or in the case of screen-based and IVRS questionnaires, as few as 14 items. To provide distraction between each of the three questionnaires (tablet, IVRS, and paper), participants completed the first and second half of the European Organization for Research and Treatment of Cancer Quality of Life Questionnaire Core-30 (EORTC QLQ-C30) on paper [19]. This distraction was incorporated into the study design to reduce the chances that participants answered the duplicate questions on different modes based on their memory of previously provided responses. The EORTC QLQ-C30 subscales are scored on a scale of 0–100 where higher functional and global health status/quality of life scores represent better function and global health status/quality of life and lower symptom scores represent a lower level of symptomology [19].

The IVRS was accessed via cell phone or land line telephone, and the paper-based questionnaire was provided on standard size pages (8.5” × 11”). The screen-based questionnaire was completed via Web-enabled touchscreen tablets. The tablets provided to the study sites had a screen size of 12.2” with the exception of one site in which the screen size was 10.5”, however this difference did not alter the presentation of the questionnaire. The screen-based version of the PRO-CTCAE is currently designed to be presented on full-size screens such as those found on large touchscreen tablets or desktop computers.

Participants were shown how to use the touchscreen tablet and IVRS by the research staff immediately prior to beginning each questionnaire. While completing each questionnaire, participants were required to answer questions without assistance from others, but could request technical assistance from research staff. Demographic and clinical variables (including comorbidities and Eastern Cooperative Oncology Group [ECOG] performance status) were reported by the clinician.

To capture the time to complete the questionnaire in each mode, research staff noted the start and end times for the paper questionnaire, the Web-based system recorded the start and end time of the tablet questionnaire, and the IVRS recorded the start and end time of each item administered by phone. The time to complete items by paper and screen-based modes of administration was calculated as the total time divided by the number of items the respondent completed. At the conclusion of the study visit, participants’ experiences with each mode were solicited via a structured exit interview conducted by the research coordinator. Participants were asked to rate whether they had any problems completing the questionnaire in each mode, using the response scale: no problems/some problems/a lot of problems. Participants were also asked their preferred mode for completing questionnaires in clinic or from home. Open-ended comments about each mode were also captured.

### Sample size

The randomized crossover design was selected because it is the most efficient, and allows for testing of order effects, mode effects, and their interactions [20]. Sample size using the formula derived by Walter (1998) [20] was based on the power to reject a null hypothesis that the intraclass correlation coefficient (ICC) between a pair of modes is less than or equal to 0.70 (*p*_0_ in the notation of Walter [1998]) using a two-sided test with α = 0.05/81; a conservative Bonferroni adjustment for three comparisons (tablet vs IVRS, tablet vs paper, and IVRS vs paper) within each of the 27 items was applied.^1^ 120 subjects would provide 18 % power to detect a true ICC of 0.80 (*p*_1_ in the notation of Walter [1998]); 79 % power for an ICC of 0.85 and >99 % power for an ICC of 0.90.

### Data analysis

Item scores were compared by mode using ICCs based on two-way analysis of variance models. The degree of mode equivalence indicated by the ICC was compared to a widely used benchmark value of ≥ 0.70 [21]. The ICCs of the across-mode comparisons including the screen-based questionnaire (i.e. tablet vs IVRS and tablet vs paper) were also compared to the test-retest reliability of the screen-based questionnaire from the validation study, in which the screen-based questionnaire was completed on consecutive business days a total of two times [3]. The use of parametric statistics (means and correlations) in the analysis of ordinal scale data is well-supported in studies with sufficiently large samples that the sampling distribution of these statistics is approximately normal [22, 23]. In simulation studies using 1000 bootstrap samples of the same sample size as this study, the sampling distributions of mean scores and correlations were approximately normally distributed, even for items with extreme floor effects (data not shown). Further, sensitivity analyses employing Wilcoxon signed-rank tests for pairwise mean rank comparisons produced results consistent with the presented results based on parametric methods (data not shown). Differences in scores by mode were evaluated with mixed-effects models for the 3-mode crossover design. The models included terms for mode, order, mode-by-order interaction, and sequence [24]. Effect sizes of the item mean differences were calculated based on Dunlop et al. 1996 [25] with bootstrapped 95 % confidence intervals; effect sizes less than 0.20 were considered acceptable [14].

Exploratory analyses were conducted to identify whether differences in scores by mode varied by participant characteristics or symptom severity. A mixed-effects model was estimated, pooling data across items; the model included terms for mode, order, mode-by-order interaction, sequence, the covariate of interest, and the mode-by-covariate interaction. Participant characteristics were gender; white vs non-white; age group (20–44, 45–64, and 65–84 years); education level; frequency of using computer to check email; physical functioning, role functioning, cognitive functioning, emotional functioning, social functioning, and global health status/quality of life, as measured by the EORTC QLQ-C30 subscales; ECOG performance status; limitations in manual dexterity due to peripheral neuropathy (average response across three modes to PRO-CTCAE item for severity of numbness or tingling in hands and feet, categorized as 0 vs. ≥1) or history of arthritis; cancer type; and current use of medications that may affect memory or cognition including chemotherapy in the past 2 weeks, opioid analgesics, sleep aids, hormone therapy, and medications for anxiety or depression. The covariate of symptom severity was defined as the PRO-CTCAE item score dichotomized as none or mild (0–1) versus moderate, severe, or very severe (2–4).

Descriptive statistics were used to summarize the time to complete items in each mode. Univariate analyses via linear regression with a single independent variable identified demographic or clinical characteristics associated with the time required to complete PRO-CTCAE items. Univariate predictors significant at the *p* < .10 level were introduced into the multivariable linear regression models using step-wise forward selection. These analyses were conducted separately for each mode of administration. Participant responses to the closed-ended questions in the structured exit interview were summarized using descriptive statistics, and any open-ended comments about each mode were summarized qualitatively.

## Results

Between February and May 2012, 112 participants completed the PRO-CTCAE questionnaire in at least one mode and 103 (92 %) completed the questionnaire in each of the three modes. Median age was 56.5 years (range 24–81 years) and 59.8 % were female (see Table 1). Self-reported race included 76.8 % white and 17 % black or African American; 9.8 % reported Hispanic/Latino ethnicity. Participants had a range of educational attainment: 53.6 % had completed at least college, 20.5 % had completed some college, 17.0 % had completed high school or GED, and 7.1 % had not completed high school. A majority (82 %) used a computer to check email or browse the internet at least several times a week. Approximately 40 % of the sample had ECOG performance status of 1 (32.1 %) or 2+ (8.9 %), reflecting some degree of functional impairment. Cancer types included: breast (34.8 %), lung/head/neck (31.3 %), gastrointestinal (11.6 %), hematological (11.6 %), and genitourinary/gynecologic (9.8 %). In the past two weeks, 62.5 % had received chemotherapy, 33.0 % had received radiation, and 0.9 % had undergone surgery. The sample was symptomatic in the past 7 days: 64 % reported experiencing pain, 75 % had fatigue, tiredness, or lack of energy, 47 % had loose or watery stools, and 49 % had nausea, each defined by a symptom score ≥ 1 as reported via the tablet questionnaire. The means and standard deviations of EORTC QLQ-C30 global health status/quality of life and functional scale scores were: Global Health Status/Quality of Life 63.8 (SD = 20.9), Physical Functioning 81.9 (SD = 20.4), Role Functioning 74.7 (SD = 27.9), Emotional Functioning 75.7 (SD = 22.3), and Social Functioning 69.7 (SD = 27.2).

**Table 1:** Patient characteristics

Characteristic	No. of Patients (*N* = 112)	Percent
Age (in years)		
Median, range	56.5 (24–81)	
Gender		
Female	67	59.8
Male	45	40.2
Ethnicity		
Hispanic or Latino	11	9.8
Not Hispanic or Latino	97	86.6
Unknown or Not reported	4	3.6
Race		
White	86	76.8
Black or African American	19	17.0
Asian	4	3.6
Native Hawaiian or Pacific Islander	2	1.8
Unknown or Not reported	1	0.9
Education		
High school or less	27	24.1
Some college	23	20.5
College graduate or more	60	53.6
Unknown or Not reported	2	1.8
Disease		
Breast	39	34.8
Lung, head or neck	35	31.3
Gastrointestinal	13	11.6
Genitourinary or Gynecologic	11	9.8
Hematological	13	11.6
Other or unknown	1	0.9
ECOG Performance Status		
0–1	102	91.1
2–4	10	8.9
Cancer treatment in the past 2 weeks		
Chemotherapy	70	62.5
Radiation	37	33.0
Surgery	1	0.9
None of the above in past 2 weeks	4	3.6
Use of computer to check e-mail or browse internet		
Never	8	7.1
Less than once a week	4	3.6
Once or several times a week	19	17.0
At least once a day	79	70.5
Unknown/Not reported	2	1.8

*Abbreviation: ECOG* Eastern Cooperative Oncology Group

The median ICCs at the item level were: tablet vs IVRS: 0.78 (range 0.55 to 0.90); tablet vs paper: 0.81 (range 0.62 to 0.96); and IVRS vs paper: 0.78 (range 0.60 to 0.91). The ICC and its 95 % confidence interval (CI) for each PRO-CTCAE item for the comparison between modes are shown in Table 2. A majority (89 %) of the ICCs were ≥0.70. Most ICCs (88 %) had a two-sided 95 % CI lower bound greater than or equal to 0.60, and 44 % of ICCs had a two-sided 95 % CI lower bound greater than or equal to 0.70. Kappa statistics of agreement for the presence/absence item rash were 0.79 for tablet vs IVRS, 0.75 for tablet vs paper, and 0.66 for IVRS vs paper (all *p* < 0.001).

**Table 2:** PRO-CTCAE item intraclass correlation by tablet, IVRS and paper modes of administration

PRO-CTCAE Item	ICC (95 % CI)			
	Mode Equivalence			Test – Retest^a^
	Tablet vs. IVRS	Tablet vs. Paper	IVRS vs. Paper	Tablet vs. Tablet
Anxiety (F)	0.77 (0.68–0.83)	0.76 (0.67–0.83)	0.72 (0.61–0.79)	0.78 (0.68–0.85)
Anxiety (S)	0.75 (0.65–0.82)	0.72 (0.62–0.80)	0.73 (0.63–0.80)	0.70 (0.58–0.80)
Anxiety (I)	0.69 (0.58–0.77)	0.74 (0.64–0.81)	0.68 (0.57–0.77)	0.83 (0.75–0.89)
Constipation (S)	0.71 (0.60–0.79)	0.76 (0.66–0.83)	0.80 (0.73–0.86)	0.83 (0.74–0.89)
Decreased appetite (S)	0.73 (0.62–0.80)	0.80 (0.72–0.86)	0.82 (0.73–0.87)	0.81 (0.71–0.87)
Decreased appetite (I)	0.78 (0.69–0.84)	0.80 (0.72–0.86)	0.70 (0.60–0.79)	0.80 (0.71–0.87)
Fatigue, tiredness, or lack of energy (S)	0.74 (0.64–0.81)	0.76 (0.67–0.83)	0.74 (0.64–0.81)	0.77 (0.66–0.85)
Fatigue, tiredness, or lack of energy (I)	0.65 (0.53–0.75)	0.70 (0.59–0.79)	0.60 (0.46–0.70)	0.79 (0.69–0.86)
Insomnia (S)	0.83 (0.76–0.88)	0.94 (0.92–0.96)	0.79 (0.71–0.85)	0.65 (0.50–0.76)
Insomnia (I)	0.76 (0.67–0.83)	0.84 (0.77–0.89)	0.81 (0.74–0.87)	0.72 (0.59–0.81)
Loose or watery stools (F)	0.80 (0.73–0.86)	0.81 (0.74–0.87)	0.76 (0.67–0.83)	0.55 (0.38–0.69)
Mouth or throat sores (S)	0.82 (0.75–0.87)	0.87 (0.82–0.91)	0.71 (0.61–0.79)	0.82 (0.73–0.88)
Mouth or throat sores (I)	0.55 (0.41–0.67)	0.62 (0.48–0.72)	0.61 (0.47–0.71)	0.81 (0.72–0.87)
Nausea (F)	0.87 (0.81–0.91)	0.90 (0.85–0.93)	0.87 (0.82–0.91)	0.79 (0.70–0.86)
Nausea (S)	0.85 (0.78–0.89)	0.87 (0.82–0.91)	0.86 (0.80–0.90)	0.84 (0.76–0.89)
Numbness/tingling in hands/feet (S)	0.89 (0.85–0.92)	0.89 (0.84–0.92)	0.86 (0.81–0.91)	0.80 (0.70–0.86)
Numbness/tingling in hands/feet (I)	0.81 (0.73–0.86)	0.85 (0.79–0.90)	0.80 (0.73–0.86)	0.55 (0.37–0.68)
Pain (F)	0.90 (0.86–0.93)	0.93 (0.90–0.95)	0.91 (0.87–0.94)	0.83 (0.75–0.89)
Pain (S)	0.88 (0.83–0.92)	0.89 (0.85–0.92)	0.86 (0.81–0.90)	0.82 (0.73–0.88)
Pain (I)	0.77 (0.68–0.84)	0.83 (0.77–0.88)	0.77 (0.68–0.83)	0.84 (0.76–0.89)
Sad or unhappy feelings (F)	0.77 (0.68–0.83)	0.80 (0.72–0.85)	0.76 (0.67–0.83)	0.73 (0.61–0.81)
Sad or unhappy feelings (S)	0.77 (0.69–0.84)	0.79 (0.71–0.85)	0.79 (0.71–0.85)	0.63 (0.48–0.75)
Sad or unhappy feelings (I)	0.69 (0.58–0.78)	0.70 (0.59–0.78)	0.72 (0.61–0.80)	0.76 (0.65–0.84)
Shortness of breath (S)	0.85 (0.79–0.89)	0.87 (0.82–0.91)	0.90 (0.85–0.93)	0.70 (0.56–0.80)
Shortness of breath (I)	0.84 (0.77–0.89)	0.73 (0.63–0.81)	0.72 (0.61–0.80)	0.66 (0.52–0.77)
Vomiting (F)	0.82 (0.75–0.87)	0.96 (0.94–0.97)	0.87 (0.82–0.91)	0.81 (0.72–0.88)
Vomiting (S)	0.82 (0.75–0.87)	0.92 (0.88–0.94)	0.83 (0.76–0.88)	0.79 (0.70–0.86)

Note: The mode equivalence of the presence/absence item assessing rash was evaluated with Kappa statistics and results are reported in the text

*Abbreviations: PRO-CTCAE* Patient-Reported Outcomes version of the Common Terminology Criteria for Adverse Events, *F* Frequency, *S* Severity, *I* Interference, *ICC* Intraclass Correlation Coefficient, *CI* Confidence Interval, *IVRS* Interactive Voice Response System

^a^Test-retest data were obtained from the validation study [3] (see Methods)

The median ICC of tablet vs tablet (test-retest reliability) for the set of items included in this mode equivalence analysis was 0.80 (range 0.55 to 0.86) [3]. The mode-equivalence ICC for tablet vs IVRS and tablet vs paper for each of the 27 items was within or above the 95 % CI of the test-retest reliability ICC for 48/54 comparisons (27/54 were within the 95 % CI and 21/54 were greater than the 95 % CI upper bound). For 6/54 comparisons the mode-equivalence ICC was below the 95 % CI lower bound of the test-retest reliability ICC.

For each PRO-CTCAE item, the median between-mode difference in the mean scores comparing tablet vs IVRS, i.e., tablet minus IVRS, was −0.04 (range −0.16 to 0.22), while for tablet vs paper it was −0.02 (range −0.11 to 0.14), and for IVRS vs paper it was 0.02 (range −0.07 to 0.19). The between-mode difference in mean scores and 95 % confidence interval around that mean for each PRO-CTCAE item is shown in Table 3. Further, the effect sizes of the differences in scores were all less than 0.20. The median effect size for the comparison of tablet vs IVRS was −0.04 (range −0.16 to 0.12), for tablet vs paper was −0.02 (range −0.11 to 0.13), and for IVRS vs paper was 0.02 (range −0.09 to 0.17). The lower and upper bounds of bootstrapped 95 % confidence intervals around the effect sizes were within +/−0.2 for 57/81 (70 %) comparisons, within +/−0.3 for 79/81 (98 %) comparisons, and within +/−0.4 for 81/81 (100 %) comparisons. Using linear mixed models, participant demographics, functioning, global health status/quality of life, or symptom severity were not associated with differences in between-mode mean scores.

**Table 3:** PRO-CTCAE Item scores by tablet, IVRS and paper modes of administration

PRO-CTCAE Item	Item Score	Difference in Item Scores by Mode		
	Mean (SD)	Mean Difference (95 % CI)		
	Tablet	Tablet – IVRS	Tablet – Paper	IVRS – Paper
Anxiety (F)	1.41 (0.90)	−0.11 (−0.24, 0.03)	−0.02 (−0.15, 0.11)	0.08 (−0.05, 0.21)
Anxiety (S)	0.62 (0.84)	−0.03 (−0.16, 0.09)	0.00 (−0.13, 0.13)	0.03 (−0.09, 0.16)
Anxiety (I)	1.20 (0.84)	−0.14 (−0.27, −0.01)	−0.06 (−0.20, 0.07)	0.08 (−0.05, 0.21)
Constipation (S)	0.80 (0.99)	−0.12 (−0.26, 0.02)	0.01 (−0.13, 0.15)	0.13 (−0.01, 0.27)
Decreased appetite (S)	0.62 (0.92)	−0.14 (−0.28, −0.01)	0.05 (−0.08, 0.18)	0.19 (0.06, 0.32)
Decreased appetite (I)	0.93 (1.03)	−0.03 (−0.16, 0.10)	0.07 (−0.06, 0.20)	0.10 (−0.04, 0.23)
Fatigue, tiredness, or lack of energy (S)	1.40 (1.17)	−0.16 (−0.30, −0.02)	−0.04 (−0.18, 0.10)	0.12 (−0.02, 0.26)
Fatigue, tiredness, or lack of energy (I)	1.53 (1.11)	−0.07 (−0.23, 0.10)	0.11 (−0.05, 0.28)	0.18 (0.02, 0.35)
Insomnia (S)	0.96 (1.19)	−0.05 (−0.17, 0.08)	0.05 (−0.08, 0.18)	0.10 (−0.03, 0.22)
Insomnia (I)	1.32 (1.22)	−0.08 (−0.22, 0.06)	−0.11 (−0.26, 0.03)	−0.03 (−0.17, 0.11)
Loose or watery stools (F)	0.79 (0.99)	−0.01 (−0.13, 0.11)	−0.01 (−0.13, 0.11)	0.00 (−0.11, 0.12)
Mouth or throat sores (S)	0.21 (0.56)	−0.03 (−0.12, 0.07)	0.01 (−0.08, 0.10)	0.04 (−0.06, 0.13)
Mouth or throat sores (I)	0.40 (0.74)	−0.01 (−0.12, 0.10)	−0.08 (−0.19, 0.04)	−0.07 (−0.18, 0.05)
Nausea (F)	0.92 (1.16)	−0.08 (−0.19, 0.03)	−0.05 (−0.15, 0.06)	0.03 (−0.07, 0.14)
Nausea (S)	0.75 (0.95)	−0.09 (−0.19, 0.01)	−0.06 (−0.17, 0.04)	0.02 (−0.08, 0.12)
Numbness/tingling in hands/feet (S)	0.70 (1.06)	−0.13 (−0.23, −0.04)	0.00 (−0.09, 0.10)	0.14 (0.04, 0.23)
Numbness/tingling in hands/feet (I)	0.89 (1.03)	0.08 (−0.04, 0.19)	0.04 (−0.08, 0.16)	−0.03 (−0.15, 0.08)
Pain (F)	1.34 (1.29)	−0.05 (−0.15, 0.06)	0.02 (−0.09, 0.13)	0.07 (−0.04, 0.17)
Pain (S)	1.06 (1.19)	0.01 (−0.10, 0.12)	−0.02 (−0.14, 0.09)	−0.03 (−0.14, 0.08)
Pain (I)	1.25 (1.20)	−0.04 (−0.19, 0.11)	−0.03 (−0.18, 0.13)	0.01 (−0.15, 0.16)
Rash (P)	0.11 (0.32)	−0.03 (−0.08, 0.02)	−0.04 (−0.09, 0.01)	−0.01 (−0.06, 0.04)
Sad or unhappy feelings (F)	1.44 (0.98)	0.12 (0.00, 0.24)	0.14 (0.02, 0.26)	0.02 (−0.10, 0.14)
Sad or unhappy feelings (S)	0.70 (0.91)	0.03 (−0.08, 0.14)	−0.01 (−0.12, 0.10)	−0.04 (−0.15, 0.07)
Sad or unhappy feelings (I)	1.11 (0.83)	0.05 (−0.08, 0.18)	0.01 (−0.12, 0.15)	−0.03 (−0.17, 0.10)
Shortness of breath (S)	0.51 (0.83)	−0.04 (−0.11, 0.04)	−0.05 (−0.12, 0.03)	−0.01 (−0.09, 0.07)
Shortness of breath (I)	0.57 (0.77)	−0.07 (−0.19, 0.05)	−0.10 (−0.22, 0.02)	−0.03 (−0.15, 0.09)
Vomiting (F)	0.29 (0.65)	−0.02 (−0.08, 0.05)	−0.02 (−0.09, 0.04)	0.00 (−0.07, 0.06)
Vomiting (S)	0.27 (0.64)	0.00 (−0.07, 0.07)	−0.06 (−0.13, 0.02)	−0.06 (−0.13, 0.02)

*Abbreviations: PRO-CTCAE* Patient-Reported Outcomes version of the Common Terminology Criteria for Adverse Events, *F* Frequency, *S* Severity, *I* Interference, *P* Presence/Absence, *SD* Standard Deviation, *CI* Confidence Interval, *IVRS* Interactive Voice Response System

The time to complete PRO-CTCAE items by mode is shown in Table 4. The average time to complete an item by Web-enabled touchscreen tablet was 11.1 seconds (SD = 8.4), by IVRS was 16.3 seconds (SD = 6.3), and by paper was 10.3 seconds (SD = 5.8). For each mode, multivariable linear regression models employing step-wise forward selection were used to identify characteristics associated with the average time to complete a PRO-CTCAE item. Time to complete items by tablet varied by age group (*b* = 2.70, *p* = 0.039); time to complete items by IVRS varied by history of arthritis (*b* = 3.48, *p* = 0.027); and time to complete items by paper varied by the following: EORTC QLQ-C30 Cognitive Functioning (*b* = −0.06*, p* = 0.016), EORTC QLQ-C30 Role Functioning (*b* = −0.05, *p* = 0.017), and age (*b* = 2.33, *p* = 0.006). These differences are very small: for example, a difference between age groups of 2.70 s per item corresponds to an additional 1.8 min to complete twenty items for people over 65 compared to people under 45. A 30 point difference in EORTC QLQ-C30 Cognitive Functioning (scored 0–100) is associated with a 0.6 min difference in completing twenty items.

**Table 4:** Time to complete PRO-CTCAE items, by mode

Mode	Number of items completed	Seconds per item	Seconds per item
	Mean (SD)	Mean (SD)	Median (25^th^ p – 75^th^ p)
Tablet	22.6 (3.4)	11.1 (8.4)	9.1 (7.4–11.6)
IVRS	22.5 (3.5)	16.3 (6.3)	15.8 (13.0–18.2)
Paper	26.9 (2.6)	10.3 (5.8)	8.6 (6.4–12.9)

*Abbreviations: PRO-CTCAE* Patient-Reported Outcomes version of the Common Terminology Criteria for Adverse Events, *IVRS* Interactive Voice Response System, *SD* Standard Deviation

The proportion of participants reporting any problems completing the PRO-CTCAE questionnaire in each mode is presented in Table 5. In the structured exit interview, 98 % reported ‘no problem’ with the paper questionnaire; 86 % had ‘no problem’ with the tablet questionnaire, and 72 % had ‘no problem’ with the IVRS phone questionnaire. 10 % of participants reported having ‘some problems’ with the tablet questionnaire. Difficulties included a slow internet connection, malfunctioning of the PRO-CTCAE system feature respondents can use to note additional symptoms, and two participants were unfamiliar with using a tablet computer. Twenty-seven percent of participants reported having ‘some problem’ with the IVRS phone questionnaire. The comments regarding IVRS revealed that some had difficulties with cell phone reception in the hospital building and therefore found it hard to hear the questions being asked via IVRS. The proportions of respondents who stated they would be comfortable using paper, tablet, and IVRS for completing a questionnaire from home were 95 %, 87 %, and 75 %, respectively. A majority of participants had a stated preference to complete questionnaires from home using the tablet (59 %), while 23 % preferred paper, 10 % preferred IVRS, and 8 % had no preference.

**Table 5:** Participant report of problems completing PRO-CTCAE items, by mode

Mode	“No problems”	“Some problems”	“A lot of problems”
Tablet	94 (86 %)	11 (10 %)	4 (4 %)
IVRS	79 (72 %)	29 (27 %)	1 (1 %)
Paper	108 (98 %)	2 (2 %)	0 (0 %)

*Abbreviations: PRO-CTCAE* Patient-Reported Outcomes version of the Common Terminology Criteria for Adverse Events, *IVRS* Interactive Voice Response System

## Discussion

This study employed a randomized crossover design to compare PRO-CTCAE item scores across three modes of data collection – Web-enabled touchscreen tablet computer, IVRS, and paper, in a large diverse U.S. sample of patients undergoing treatment for cancer. In summary, the mode-equivalence of items was moderate to high, and similar to test-retest reliability. Differences in mean scores by mode were generally trivial in size, and were not moderated by clinical or demographic characteristics, including gender, education, race/ethnicity or symptom severity. This study was designed to identify large differences between modes; employing stricter criteria (that is, requiring that the lower bound of the 95 % CI around the ICC be greater than 0.70 for true ICCs below 0.85) would have made the necessary sample size infeasible. Although the study was not powered to identify ICCs below 0.85 as being statistically greater than 0.70, the observed point estimates of between-mode ICCs and very small mean differences provide evidence to support study designs that employ multiple modes of administration.

The equivalence of PRO-CTCAE scores by mode observed here is consistent with the findings of mode-equivalence studies of other PRO measures commonly used in cancer. A mode equivalence study of the EORTC QLQ-C30 which examined the equivalence of multi-item subscales across screen-based, IVRS and paper found ICCs ranged from 0.79 to 0.90 with 95 % lower confidence intervals greater than 0.70 [7]. A mode equivalence study of the Patient-Reported Outcomes Measurement Information System® (PROMIS®) adult measures of physical function, fatigue, and depression, which compared personal computer administration with IVRS, paper, and personal digital assistant in a randomized cross-over design, observed ICCs ranging from 0.85 to 0.94 and no evidence of differences in score level [8]. The EORTC QLQ-C30 and PROMIS® short forms were evaluated at the level of multi-item scales, whereas the PRO-CTCAE is composed of individual items that are not combined into scale scores. Scales with a small number of items will tend to have lower measurement reliability and similarly, the ICC of the between-mode comparisons will also be lower [13, 26]. However, given that symptomatic adverse event reporting – the purpose of the PRO-CTCAE, generally requires surveillance on a wide range of toxicities at frequent intervals, longer questionnaires would produce unacceptable respondent burden.

The design and sampling plan of this mode equivalence study had a number of strengths. Data were collected from a diverse sample of U.S. cancer patients, reflecting a range of race/ethnicity (22.4 % were non-white), education level (44.6 % did not have a college degree), adult ages, treatment settings, and cancer types, and the sampling was enriched for patients with poor performance status who were symptomatic. The randomized factorial design employed in the data collection enabled direct comparisons of responses by mode within patient. The study was successful in achieving a high rate of questionnaire completion for all three modes (92 % completed all three modes). This was one of the anticipated benefits of having the questionnaires completed in one study visit. Most importantly, there would also be no change in health status between assessments. A distractor questionnaire, one page of the EORTC QLQ-C30, was employed in the study design between modes so that respondents would not be completing the PRO-CTCAE questionnaires one directly after the other. The inclusion of the EORTC QLQ-C30 functional and health status/QOL subscales and comprehensive clinical data including current medications and treatment, also provided the opportunity to evaluate several important hypothesized covariates in the analysis of scores by mode and the time to complete each mode.

Three caveats must be considered in interpreting our findings about the mode equivalence of PRO-CTCAE items. First, it is possible that despite the use of distractor questionnaires between modes, participants recalled their responses to the previous set of questions. In between assessments, the participant completed the distraction task and was oriented to using the next mode, which took approximately 10 min. This study was designed so that assessments were completed on the same day in order to avoid differences in scores being due to changes in symptomology, and it was not feasible for assessments to be completed several hours apart because that would have significantly extended participants’ study visits, thus imposing an unacceptable level of burden in patients undergoing active cancer treatment. Second, comparisons of the between-mode reliability statistics and test-retest reliability statistics must consider that the test-retest reliability was based on assessments gathered approximately 1–3 days apart, whereas all three mode equivalence assessments were gathered within a 1 hour period when comparatively little fluctuation in symptom severity would be expected, and that the 95 % CI of the test-retest reliability is dependent on the sample size. In addition, the between-mode and the within-mode (test-retest) reliability estimates were derived in different samples, though both were drawn from the same study population in terms of the eligibility criteria and recruitment strategy. Third, an unavoidable limitation of statistical estimates of between-mode differences is that the agreement of two assessments depends on the distribution of symptoms scores in the patient sample. Prior studies have found higher levels of agreement between ratings when both assessments are “0” (symptom is absent) but lower levels of agreement in the upper ranges of the severity scale [27]. Therefore the level of agreement or reliability between two assessments may be higher when a larger proportion of the sample does not have the symptom in question. However, it is a strength of our study that approximately half of respondents were experiencing common cancer symptoms, including pain, fatigue, diarrhea, and nausea.

Across all three modes, PRO-CTCAE items were completed rapidly. It should be noted that estimation of the total time to complete a questionnaire by each mode depends upon the number of items presented to the respondent. Further, because paper questionnaires do not incorporate conditional branching or skip patterns, as do screen-based and IVRS questionnaires, a participant completing a paper questionnaire would generally have to complete more items. For example, in a 28-item questionnaire with conditional branching, a respondent may only complete 20 items. Thus, because the conditional branching present when the questionnaire is completed by electronic modes leads to variation in the number of items completed by the respondent, we estimated the time to complete a certain number of items, rather than the time to complete a questionnaire that may contain a varying number of items. Based on our study, we estimate that completing twenty PRO-CTCAE items would take on average 3.4 min by paper, 3.7 min by Web-enabled touchscreen tablet computer, and 5.4 min by IVRS. As an example of an estimate of respondent burden for human subjects research applications, 75 % of the sample would complete twenty items in 4.3 min by paper, in 3.8 min by Web-enabled touchscreen tablet computer, and in 6.1 min by IVRS. There was no evidence of clinically meaningful variation in completion times by participant characteristics, including impairments in physical or cognitive functioning. Our findings suggest that completion of PRO-CTCAE items is generally not laborious, even for those respondents who may have some degree of functional limitation.

The proportions of respondents who stated that they would be comfortable using paper, Web-enabled touchscreen tablet, and IVRS for completing a questionnaire from home were 95 %, 87 %, and 75 %, respectively. Further, 98 % reported ‘no problem’ with the paper questionnaire; 86 % had ‘no problem’ with Web-enabled touchscreen tablet, and 72 % had ‘no problem’ with the IVRS questionnaire. Some study participants experienced technical difficulties with cell phone reception and Wi-Fi-based computer connections within the clinics where the data collection took place.^2^ Because of the size and construction of many large institutional medical buildings, connectivity issues may be a key consideration for in-clinic PRO data collection. It is likely that the participant preferences for each mode reported in this study were influenced in part by technical issues experienced in our participating clinics. These stated preferences may not be generalizable to at-home reporting or clinic settings without connectivity issues.

The rate of missing data in this study was extremely low in part because questionnaires were completed in clinic as part of a study visit. The potential for missing data when questionnaires are completed outside the clinic setting, including the potential for variable rates of missing data across modes, should be considered in the design and implementation of cancer clinical trials that employ PRO-CTCAE to collect symptomatic adverse events.

## Conclusion

Our study results describe the equivalence of PRO-CTCAE across three modes of data collection both within- and between-participants, and the findings are consistent with other studies examining the mode equivalence of other PRO measures. We observed moderate to high levels of agreement across modes, and provide evidence of the acceptability of paper, Web-enabled touchscreen tablet, and IVRS modes of administration to a majority of respondents. Although the study was powered to detect moderate or larger discrepancies between modes, these results support study designs that are responsive to varying patient or investigator preference for mode of administration, and justify pooled analyses or comparison of results across studies that employ different PRO-CTCAE modes of administration.

## Abbreviations

CI: Confidence Interval
CTCAE: Common Terminology Criteria for Adverse Events
ECOG: Eastern Cooperative Oncology Group
EORTC QLQ-C30: European Organization for Research and Treatment of Cancer Quality of Life Questionnaire Core-30
F: Frequency
I: Interference
ICC: Intraclass correlation coefficients
IVRS: Interactive Voice Response System
NCCCP: NCI Community Cancer Centers Program
NCI: National Cancer Institute
P: Presence/Absence
PRO: patient-reported outcome
PRO-CTCAE: Patient-Reported Outcomes version of the Common Terminology Criteria for Adverse Events
PROMIS®: Patient-Reported Outcomes Measurement Information System®
S: Severity
SD: Standard Deviation

## Footnotes

1 One additional item, measuring the presence of rash (yes/no) was assessed using Kappa statistics of agreement and was not included in this count.

2 To address difficulties in hearing the recorded questions, particularly in a clinic where there can be a lot of background noise, technical adjustments have been made to improve the fidelity of the audio component of the PRO-CTCAE IVRS.
